# 1-(Butan-2-yl­idene)-2-(2-nitro­phen­yl)hydrazine

**DOI:** 10.1107/S1600536809031420

**Published:** 2009-08-15

**Authors:** Heng-yu Qian, Zhi-gang Yin, Zhi-qiang Yao

**Affiliations:** a Key Laboratory of Surface and Interface Science of Henan School of Materials & Chemical Engineering, Zhengzhou University of Light Industry, Zhengzhou 450002, People’s Republic of China

## Abstract

Crystals of the title compound, C_10_H_13_N_3_O_2_, were obtained from a condensation reaction of butan-2-one and 1-(2-nitro­phen­yl)hydrazine. The mol­ecule exhibits a nearly coplanar structure, except for the methyl and methyl­ene H atoms, the largest deviations from the mean plane defined by all non-H atoms, except for the nitro group, being 0.120 (2) Å for one of the nitro O atoms. Intra­molecular N—H⋯O hydrogen bonding helps to establish the mol­ecular configuration.

## Related literature

For applications of Schiff base compounds, see: Kahwa *et al.* (1986 ▶); Santos *et al.* (2001 ▶).
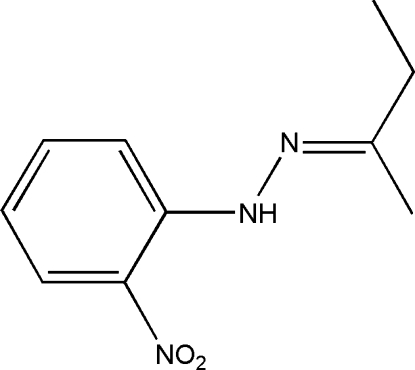


## Experimental

### 

#### Crystal data

C_10_H_13_N_3_O_2_
*M* *_r_* = 207.23
Monoclinic, 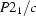

*a* = 7.3079 (11) Å
*b* = 10.2150 (17) Å
*c* = 14.763 (2) Å
β = 100.058 (9)°
*V* = 1085.1 (3) Å^3^
*Z* = 4
Mo *K*α radiation
μ = 0.09 mm^−1^
*T* = 296 K
0.28 × 0.21 × 0.11 mm

#### Data collection

Bruker SMART CCD area-detector diffractometer
Absorption correction: none
7304 measured reflections
2116 independent reflections
1099 reflections with *I* > 2σ(*I*)
*R*_int_ = 0.024

#### Refinement

*R*[*F* ^2^ > 2σ(*F* ^2^)] = 0.047
*wR*(*F* ^2^) = 0.159
*S* = 0.93
2116 reflections
141 parameters
1 restraint
H atoms treated by a mixture of independent and constrained refinement
Δρ_max_ = 0.17 e Å^−3^
Δρ_min_ = −0.15 e Å^−3^

### 

Data collection: *SMART* (Bruker, 1998 ▶); cell refinement: *SAINT* (Bruker, 1998 ▶); data reduction: *SAINT*; program(s) used to solve structure: *SHELXS97* (Sheldrick, 2008 ▶); program(s) used to refine structure: *SHELXL97* (Sheldrick, 2008 ▶); molecular graphics: *ORTEP-3 for Windows* (Farrugia, 1997 ▶); software used to prepare material for publication: *WinGX* (Farrugia, 1999 ▶).

## Supplementary Material

Crystal structure: contains datablocks global, I. DOI: 10.1107/S1600536809031420/xu2581sup1.cif

Structure factors: contains datablocks I. DOI: 10.1107/S1600536809031420/xu2581Isup2.hkl

Additional supplementary materials:  crystallographic information; 3D view; checkCIF report

## Figures and Tables

**Table 1:** Hydrogen-bond geometry (Å, °)

*D*—H⋯*A*	*D*—H	H⋯*A*	*D*⋯*A*	*D*—H⋯*A*
N2—H2*A*⋯O2	0.889 (16)	1.969 (16)	2.604 (3)	127.2 (14)

**Fig. 1.**
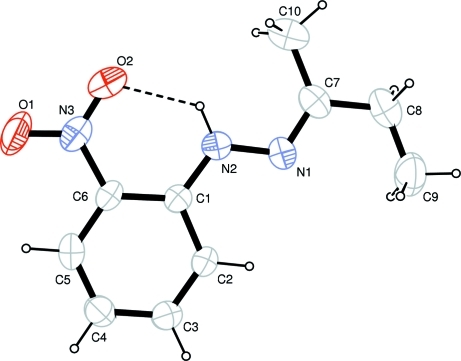
The molecular structure of the title compound. Displacement ellipsoids are drawn at the 30% probability level. Hydrogen bonding is shown in dashed line.

## supplementary crystallographic information

### Comment

The chemistry of Schiff base has attracted a great deal of interest in recent years. These compounds play an important role in the development of various proteins and enzymes (Kahwa *et al.*, 1986; Santos *et al.*, 2001). As part of our in the study of the coordination chemistry of Schiff bases, we synthesized the title compound and determined its crystal structure.

The molecular structure of (I) is shown in Fig. 1. The molecules is roughly planar, with the largest deviations from the mean plane defined by all non-H atoms, except the nitro group, being -0.120 (2) for atom O2.

Intramolecular N—H···O hydrogen bond is observed in compound (I), and this helps to stabilize the configuration of the molecule.

### Experimental

2-Nitrophenylhydrazine (1 mmol, 0.153 g) was dissolved in anhydrous ethanol (15 ml). The mixture was stirred for several min at 351 K, then butan-2-one (1 mmol, 0.72 g) in ethanol (8 ml) was added dropwise and the mixture was stirred at refluxing temperature for 2 h. The product was isolated and recrystallized from methanol, red single crystals were obtained after 3 d.

### Refinement

Imino H atom was located in a difference Fourier map and positional parameters were refined with a fixed isotropic thermal parameter of 0.08 Å^2^. Other H atoms were positioned geometrically and refined as riding with C—H = 0.93 (aromatic), 0.97 (methylene) and 0.96 Å (methyl), with U_iso_(H) = 1.5U_eq_(C) for methyl H atoms and 1.2U_eq_(C) for the others.

### Crystal data

**Table:**

C_10_H_13_N_3_O_2_	*F*(000) = 440
*M**_r_* = 207.23	*D*_x_ = 1.268 Mg m^−^^3^
Monoclinic, *P*2_1_/*c*	Mo *K*α radiation, λ = 0.71073 Å
Hall symbol: -P 2ybc	Cell parameters from 1572 reflections
*a* = 7.3079 (11) Å	θ = 2.4–26.0°
*b* = 10.2150 (17) Å	µ = 0.09 mm^−^^1^
*c* = 14.763 (2) Å	*T* = 296 K
β = 100.058 (9)°	Block, red
*V* = 1085.1 (3) Å^3^	0.28 × 0.21 × 0.11 mm
*Z* = 4	

### Data collection

**Table:**

Bruker SMART CCD area-detector diffractometer	1099 reflections with *I* > 2σ(*I*)
Radiation source: fine-focus sealed tube	*R*_int_ = 0.024
graphite	θ_max_ = 26.0°, θ_min_ = 2.4°
ω scans	*h* = −9→8
7304 measured reflections	*k* = −11→12
2116 independent reflections	*l* = −13→18

### Refinement

**Table:**

Refinement on *F*^2^	Primary atom site location: structure-invariant direct methods
Least-squares matrix: full	Secondary atom site location: difference Fourier map
*R*[*F*^2^ > 2σ(*F*^2^)] = 0.047	Hydrogen site location: inferred from neighbouring sites
*wR*(*F*^2^) = 0.159	H atoms treated by a mixture of independent and constrained refinement
*S* = 0.93	*w* = 1/[σ^2^(*F*_o_^2^) + (0.0917*P*)^2^] where *P* = (*F*_o_^2^ + 2*F*_c_^2^)/3
2116 reflections	(Δ/σ)_max_ = 0.010
141 parameters	Δρ_max_ = 0.17 e Å^−^^3^
1 restraint	Δρ_min_ = −0.14 e Å^−^^3^

### Special details

**Table:**

Geometry. All e.s.d.'s (except the e.s.d. in the dihedral angle between two l.s. planes) are estimated using the full covariance matrix. The cell e.s.d.'s are taken into account individually in the estimation of e.s.d.'s in distances, angles and torsion angles; correlations between e.s.d.'s in cell parameters are only used when they are defined by crystal symmetry. An approximate (isotropic) treatment of cell e.s.d.'s is used for estimating e.s.d.'s involving l.s. planes.
Refinement. Refinement of *F*^2^ against ALL reflections. The weighted *R*-factor *wR* and goodness of fit *S* are based on *F*^2^, conventional *R*-factors *R* are based on *F*, with *F* set to zero for negative *F*^2^. The threshold expression of *F*^2^ > σ(*F*^2^) is used only for calculating *R*-factors(gt) *etc*. and is not relevant to the choice of reflections for refinement. *R*-factors based on *F*^2^ are statistically about twice as large as those based on *F*, and *R*- factors based on ALL data will be even larger.

### Fractional atomic coordinates and isotropic or equivalent isotropic displacement parameters (Å^2^)

**Table:**

	*x*	*y*	*z*	*U*_iso_*/*U*_eq_	
N1	0.3024 (2)	1.04656 (17)	0.09960 (11)	0.0674 (5)	
C1	0.2088 (2)	0.89169 (19)	−0.01835 (13)	0.0551 (5)	
N2	0.2496 (2)	1.01728 (17)	0.00803 (12)	0.0678 (5)	
C2	0.2183 (3)	0.79113 (19)	0.04780 (12)	0.0621 (6)	
H2	0.2495	0.8122	0.1099	0.075*	
C6	0.1570 (2)	0.85236 (19)	−0.11104 (12)	0.0587 (5)	
C5	0.1221 (3)	0.7221 (2)	−0.13432 (14)	0.0690 (6)	
H5	0.0889	0.6985	−0.1959	0.083*	
N3	0.1382 (3)	0.9459 (2)	−0.18488 (14)	0.0817 (6)	
C3	0.1830 (3)	0.66460 (19)	0.02310 (14)	0.0698 (6)	
H3	0.1907	0.6007	0.0685	0.084*	
C4	0.1358 (3)	0.6288 (2)	−0.06817 (15)	0.0732 (6)	
H4	0.1136	0.5415	−0.0841	0.088*	
O2	0.1590 (3)	1.0630 (2)	−0.16759 (12)	0.1089 (7)	
C7	0.3445 (3)	1.1663 (2)	0.11902 (16)	0.0713 (6)	
O1	0.1019 (3)	0.90665 (19)	−0.26427 (11)	0.1204 (7)	
C8	0.4002 (3)	1.2013 (2)	0.21782 (18)	0.0938 (8)	
H8A	0.5240	1.2387	0.2266	0.113*	
H8B	0.3166	1.2687	0.2325	0.113*	
C10	0.3419 (3)	1.2749 (2)	0.05076 (18)	0.0974 (8)	
H10A	0.4223	1.2531	0.0080	0.146*	
H10B	0.3843	1.3543	0.0824	0.146*	
H10C	0.2175	1.2869	0.0180	0.146*	
C9	0.4004 (4)	1.0931 (3)	0.28360 (19)	0.1200 (10)	
H9A	0.2803	1.0523	0.2740	0.180*	
H9B	0.4286	1.1266	0.3452	0.180*	
H9C	0.4926	1.0298	0.2745	0.180*	
H2A	0.240 (3)	1.0806 (15)	−0.0338 (11)	0.080*	

### Atomic displacement parameters (Å^2^)

**Table:**

	*U*^11^	*U*^22^	*U*^33^	*U*^12^	*U*^13^	*U*^23^
N1	0.0685 (11)	0.0696 (12)	0.0636 (12)	−0.0003 (9)	0.0106 (8)	−0.0109 (9)
C1	0.0509 (11)	0.0590 (12)	0.0556 (12)	0.0053 (9)	0.0102 (8)	0.0030 (10)
N2	0.0764 (12)	0.0608 (12)	0.0652 (13)	0.0025 (9)	0.0095 (9)	0.0037 (8)
C2	0.0672 (13)	0.0693 (14)	0.0485 (11)	0.0010 (10)	0.0062 (9)	0.0024 (10)
C6	0.0557 (11)	0.0734 (14)	0.0468 (11)	0.0076 (10)	0.0081 (8)	0.0083 (10)
C5	0.0601 (13)	0.0917 (17)	0.0549 (12)	−0.0007 (11)	0.0087 (10)	−0.0156 (12)
N3	0.0826 (13)	0.1009 (16)	0.0613 (13)	0.0115 (11)	0.0116 (9)	0.0134 (12)
C3	0.0758 (15)	0.0658 (14)	0.0680 (14)	−0.0028 (11)	0.0126 (11)	0.0065 (11)
C4	0.0764 (15)	0.0667 (14)	0.0777 (16)	−0.0052 (11)	0.0169 (12)	−0.0077 (12)
O2	0.1446 (17)	0.0932 (13)	0.0870 (13)	0.0062 (12)	0.0148 (11)	0.0327 (10)
C7	0.0523 (12)	0.0700 (15)	0.0934 (17)	0.0034 (11)	0.0173 (11)	−0.0162 (13)
O1	0.1572 (18)	0.1516 (17)	0.0490 (11)	0.0180 (13)	0.0086 (10)	0.0100 (10)
C8	0.0826 (16)	0.0953 (18)	0.106 (2)	−0.0056 (13)	0.0236 (14)	−0.0338 (16)
C10	0.0861 (18)	0.0704 (16)	0.136 (2)	0.0024 (13)	0.0191 (15)	0.0039 (15)
C9	0.135 (2)	0.144 (3)	0.0799 (17)	−0.036 (2)	0.0144 (16)	−0.0224 (18)

### Geometric parameters (Å, °)

**Table:**

N1—C7	1.282 (2)	C3—C4	1.380 (3)
N1—N2	1.372 (2)	C3—H3	0.9300
C1—N2	1.359 (2)	C4—H4	0.9300
C1—C6	1.413 (3)	C7—C10	1.496 (3)
C1—C2	1.411 (2)	C7—C8	1.488 (3)
N2—H2A	0.888 (9)	C8—C9	1.471 (3)
C2—C3	1.355 (2)	C8—H8A	0.9700
C2—H2	0.9300	C8—H8B	0.9700
C6—C5	1.387 (3)	C10—H10A	0.9600
C6—N3	1.438 (2)	C10—H10B	0.9600
C5—C4	1.356 (3)	C10—H10C	0.9600
C5—H5	0.9300	C9—H9A	0.9600
N3—O1	1.223 (2)	C9—H9B	0.9600
N3—O2	1.227 (2)	C9—H9C	0.9600
			
C7—N1—N2	116.33 (18)	C5—C4—H4	120.3
N2—C1—C6	123.67 (17)	C3—C4—H4	120.3
N2—C1—C2	120.49 (18)	N1—C7—C10	125.6 (2)
C6—C1—C2	115.84 (18)	N1—C7—C8	117.5 (2)
C1—N2—N1	119.87 (16)	C10—C7—C8	116.8 (2)
C1—N2—H2A	120.1 (14)	C9—C8—C7	115.8 (2)
N1—N2—H2A	120.0 (14)	C9—C8—H8A	108.3
C3—C2—C1	121.61 (18)	C7—C8—H8A	108.3
C3—C2—H2	119.2	C9—C8—H8B	108.3
C1—C2—H2	119.2	C7—C8—H8B	108.3
C5—C6—C1	121.30 (17)	H8A—C8—H8B	107.4
C5—C6—N3	117.42 (19)	C7—C10—H10A	109.5
C1—C6—N3	121.28 (19)	C7—C10—H10B	109.5
C4—C5—C6	120.57 (19)	H10A—C10—H10B	109.5
C4—C5—H5	119.7	C7—C10—H10C	109.5
C6—C5—H5	119.7	H10A—C10—H10C	109.5
O1—N3—O2	121.1 (2)	H10B—C10—H10C	109.5
O1—N3—C6	119.0 (2)	C8—C9—H9A	109.5
O2—N3—C6	119.9 (2)	C8—C9—H9B	109.5
C2—C3—C4	121.22 (18)	H9A—C9—H9B	109.5
C2—C3—H3	119.4	C8—C9—H9C	109.5
C4—C3—H3	119.4	H9A—C9—H9C	109.5
C5—C4—C3	119.5 (2)	H9B—C9—H9C	109.5

### Hydrogen-bond geometry (Å, °)

**Table:**

*D*—H···*A*	*D*—H	H···*A*	*D*···*A*	*D*—H···*A*
N2—H2A···O2	0.89 (2)	1.97 (2)	2.604 (3)	127 (1)
